# Clinical Features of Patients Undergoing the Head-Up Tilt Test and Its Safety and Efficacy in Diagnosing Vasovagal Syncope in 4,873 Patients

**DOI:** 10.3389/fcvm.2021.781157

**Published:** 2022-01-12

**Authors:** Lingping Xu, Xiangqi Cao, Rui Wang, Yichao Duan, Ye Yang, Junlong Hou, Jing Wang, Bin Chen, Xianjun Xue, Bo Zhang, Hua Ma, Chaofeng Sun, Fengwei Guo

**Affiliations:** ^1^Department of Cardiovascular Medicine, The First Affiliated Hospital of Xi'an Jiaotong University, Xi'an, China; ^2^Department of Cardiovascular Medicine, The Xianyang Central Hospital, Xianyang, China; ^3^Stroke Centre and Department of Neurology, The First Affiliated Hospital of Xi'an Jiaotong University, Xi'an, China; ^4^Department of Cardiovascular Surgery, The First Affiliated Hospital of Xi'an Jiaotong University, Xi'an, China

**Keywords:** neurocardiovascular, diagnosis, drug-potentiated head-up tilt test, syncope, vasovagal syncope, heart rate, blood pressure

## Abstract

**Background:** The head-up tilt test (HUTT) is a useful diagnostic tool in patients with suspected vasovagal syncope (VVS).

**Objectives:** We aimed to investigate the direct drug-potentiated HUTT in patients with recurrent syncope or precursor syncope and to assess the diagnostic value of the direct drug-potentiated HUTT.

**Methods:** The medical history and direct drug-potentiated HUTT records of patients who complained of syncope or precursor syncope and who visited The Xianyang Central Hospital from January 2016 to December 2020 were retrospectively reviewed.

**Results:** A total of 4,873 patients (age = 43.8 ± 17.6 years; male = 2,064 [42.4%]) were enrolled in our study. Overall, 2,343 (48.1%) showed positive responses as follows: 1,260 (25.9%) with the mixed type, 34 (0.7%) with the cardioinhibitory type, 580 (11.9%) with the vasodepressor type, 179 (3.7%) with postural tachycardia syndrome (POTS), and 290 (6.0%) with orthostatic hypotension (OH). The study showed that prior to syncope or near-syncope symptoms, patients first presented an increase in heart rate (HR), followed by decreases in blood pressure (BP) and HR successively. Among the patients in the syncope group, the sensitivity of the HUTT was 65.9%, which was significantly higher than a sensitivity of 44.8% for patients in the non-syncope group (*P* < 0.01). The sensitivity of the HUTT was higher for females than males in both the syncope group (52.6% in males and 77.9% in females, *P* < 0.01) and the non-syncope group (36.5% in males and 50.6% in females, *P* < 0.01). Within the four age groups (<20, 21–40, 41–60, and >60 years old), the sensitivities were 74.7%, 67.7%, 45.6%, and 31.2%, respectively. And all gender, age and symptom (whether suffered from a syncope or not) significantly affected the positive responses of HUTT. There were two adverse events and no deaths during the HUTT in this study.

**Conclusion:** The direct drug-potentiated HUTT is a safe and highly sensitive tool with which to diagnose VVS. Patients with precursor syncope symptoms without syncope should undergo a HUTT, especially young females presenting with weakness and sweating, which can decrease the probability of a misdiagnosis or a missed diagnosis.

## Introduction

Vasovagal syncope (VVS) is one of the most common causes of convulsive syncope, which accounts for 50% of all syncope cases, and is characterized by a transient loss of consciousness and a cascade of associated symptoms (1, 2). The pathophysiology of VVS is not completely understood, but it seems to be derived from a neurocardiogenic reflex-mediated inhibition of sympathetic activity, the overexcitation of parasympathetic activity, heart rate (HR) deceleration, and hypotension resulting in sudden peripheral and cerebral hypoperfusion due to a variety of precipitating factors, such as orthostatic (upright) stress, pain, or emotional triggers (3, 4).

The head-up tilt test (HUTT), as a class IIa recommendation, is widely used and has remained a practical tool in the diagnosis and management of VVS (5, 6). A previous study showed that patients with VVS had many autonomic symptoms, such as nausea, vomiting, abdominal discomfort, pallor, sweating and palpitations, yawning, stridor, salivation, pupillary dilatation, and urinary incontinence, in addition to syncope. Additionally, other symptoms related to cerebral and retinal hypoperfusion were commonly observed in VVS patients, such as dizziness, light-headedness, and blurred vision (7). Therefore, patients presenting with the symptoms mentioned above should be evaluated for VVS. To enrich the relevant data, we performed the HUTT on 4,873 patients from January 2016 to December 2020 at The Xianyang Central Hospital, and we aimed to explore the application of the direct drug-potentiated HUTT in patients with a complaint of recurrent or precursor syncope.

## Study Population and Methods

### Study Population and Data Collection

The study population consisted of all patients undergoing a HUTT due to recurrent unexplained syncope or precursor syncope from January 2016 to December 2020 at The Xianyang Central Hospital. The symptoms of precursor syncope were identified as dizziness, light-headedness, blurred vision, nausea, vomiting, pallor, diaphoresis, chest tightness. and palpitations. We mainly excluded patients who had a history of cardiomyopathies, intracranial disease, seizure, psychogenic pseudosyncope, carotid sinus syndrome, and any HUTT-associated contraindication as determined by a thorough evaluation, including a careful medical history, physical examination, electrocardiogram (ECG)/Holter ECG, echocardiography, chest X-ray, electroencephalogram, and brain computed tomography (CT)/magnetic resonance imaging (MRI). Patients who took any medications that impacted the autonomic nervous system or circulatory system were also excluded from the analysis. The study complied with the Helsinki Declaration and was approved by the Institutional Ethics Committee of The Xianyang Central Hospital (No. 282000010). All patients provided their or their parents' written informed consent.

### HUTT Protocol

The HUTT was performed using an electrically controlled tilt table (SHUT-100), and the blood pressures (BP) and HR of the patients were continuously monitored. The direct drug-potentiated HUTT was performed without a basic HUTT stage. We used a protocol that included 5–10 min of rest in the supine position, after which 500 μg (4–6 μg/kg for children) of nitroglycerin was administered sublingually, followed by a phase of 20 min with the patient's head positioned with a tilt of 60–70 degrees (patients ≤ 18 or ≥75 years old underwent a 60-degree HUTT, and the other patients underwent a 70-degree HUTT). The HUTT was completed if a positive response occurred or after 20 min. After 30 min of rest, patients with a negative response who were highly suspected of having VVS underwent an isoprenaline-stimulated HUTT, and the patients spent another 20 min with their head tilted up to the same degree. They were initially treated with isoprenaline 1 μg/min as an intravenous drip, which was increased by 1 μg/min at 5-min intervals, up to a maximum of 3 μg/min. The HUTT with isoprenaline infusion was halted when a positive response occurred, the average HR increased by 20–25% from the baseline value, or the fastest HR was over 150 beats per min (bpm) at 20 min (8).

Positive responses of the patients were due to VVS, postural tachycardia syndrome (POTS), and orthostatic hypotension (OH). Vasovagal syncope was defined as syncope or near-syncope, simultaneously accompanied by the following hemodynamic changes: a BP decrease [systolic BP (SBP) ≤ 80 mmHg and/or a diastolic BP (DBP) ≤ 50 mmHg or a mean arterial pressure decrease ≥25%] and/or bradycardia (HR <50 bpm) or cardiac arrest >3 s (9–11). Vasovagal syncope was further classified into three responses based on the classification of the Vasovagal Syncope International Study (VASIS) (12): mixed type (VASIS I), cardioinhibitory type (VASIS II), and vasodepressor type (VASIS III). Postural tachycardia syndrome (except OH) was identified as an HR increase of >30 bpm or an HR ≥120 bpm if the BP did not decrease significantly (the decrease was <20/10 mmHg) (10). Orthostatic hypotension was defined by an SBP decrease of ≥20 mmHg and/or a DBP decrease of ≥10 mmHg (within the first 3 min of the HUTT) if the HR did not significantly decrease (11). The criteria of positive HUTT in the study were coincident for both non-syncope and syncope patients.

Finally, the patients were mainly classified based on the different clinical symptoms that were presented at the time of referral, and the patients were classified into two groups (the syncope group and non-syncope group). In the non-syncope group, the patients were mainly classified into five subgroups, namely, the dizziness group, chest tightness/palpitation group, intermittent weakness group, sweating group, and intermittent unexplained arrhythmia group. In addition, all patients were divided into four groups (<20, 21–40, 41–60, and > 60 years old) based on age. The positive rate of the HUTT and the percentage of each positive class were compared within groups or subgroups.

### Statistical Analysis

Statistical analysis was performed using SPSS 22.0. Continuous variables are presented as the mean ± standard deviation (SD) for normally distributed data and were compared using Student's *t*-test. Medians (P25, P75) were used to describe the non-normally distributed continuous variables and were compared using the Mann-Whitney U-test. Categorical data are presented as frequencies (percentages) and were compared using the χ^2^-test. Binary logistic regression was used to analyze the related factors for positive HUTT results. A *p*-value of < 0.05 was considered statistically significant.

## Results

### Baseline Characteristics

A total of 4,873 patients who underwent the HUTT due to syncope or near-syncope were included in the study. The mean age at referral was 48.3 ± 17.6 years, and 2,064 (42.4%) patients were males. There were 768 patients (365 [47.5%] males) aged 41.6 ± 19.2 years in the syncope group and 4,105 patients (1,699 [41.4%] males) aged 49.6 ± 17.0 years in the non-syncope group. Patients in the syncope group were younger than the patients in the non-syncope group (*P* < 0.001). The proportion of males in the syncope group was higher than that in the non-syncope group (*P* = 0.002). The common symptoms at referral were syncope (15.8%), dizziness (60.7%), chest tightness/palpitation (21.0%), weakness (3.1%), sweating (1.9%), and intermittent unexplained arrhythmia (3.3%). The first department in which the patients chose to be examined was the Department of Neurology (50.4%), followed by the Department of Cardiology (35.9%), and other departments (13.7%), including the Departments of Ophthalmology and Otorhinolaryngology, Orthopedics and Psychiatry in the syncope group, and the Department of Neurology (49.4%), the Department of Cardiology (43.2%), and other departments (8.3%) in the non-syncope group. Compared with the non-syncope group, the proportion of patients who initially visited other departments in the syncope group was higher (*P* < 0.001) (Table 1).

**Table 1:** Basic clinical features and HUTT results of the patients.

**Parameter**	**Syncope group (*n* = 768)**	**Nonsyncope group (*n* = 4,105)**	***P*-value**
Age (years)	41.6 ± 19.2	49.6 ± 17.0	<0.001
Male	365 (47.5)	1,699 (41.4)	0.002
**First consultation department**			
Department of Cardiology	276 (35.9)	1,738 (42.3)	0.001
Department of Neurology	387 (50.4)	2,026 (49.4)	0.598
Other departments	105 (13.7)	341 (8.3)	<0.001
Positive HUTT	506 (65.9)	1,837 (44.8)	<0.001
**Positive types of HUTT**			
Mixed type	331 (43.1)	929 (22.6)	<0.001
Cardioinhibitory type	9 (1.2)	25 (0.6)	0.085
Vasodepressor type	107 (13.9)	473 (11.5)	0.058
POTS	26 (3.4)	153 (3.7)	0.644
OH	33 (4.3)	257 (6.3)	0.035
Decrease in SBP (mmHg)	43.8 ± 18.7	42.8 ± 18.5	0.707
Decrease in HR (bpm)	44.7 ± 18.0	40.7 ± 18.3	0.782

*HUTT, head-up tilt test; POST, postural tachycardia syndrome; OH, orthostatic hypotension; SBP, systolic blood pressure; HR, heart rate; bpm, beats per min*.

### HUTT Results

Among all 4,873 patients, 2,343 (48.1%) exhibited a positive response (1,260 [25.9%] with the mixed type, 34 [0.7%] with the cardioinhibitory type, 580 [11.9%] with the vasodepressor type, 179 [3.7%] with POTS, 290 [6.0%] with OH), and 2,530 (51.9%) patients exhibited a negative reaction. Of the 768 patients with syncope, a positive response was observed in 506 (65.9%) patients, including mixed type in 331 (43.1%) cases, cardioinhibitory type in 9 (1.2%) cases, vasodepressor type in 107 (13.9%) cases, POTS in 26 (3.4%) cases, and OH in 33 (5.1%) cases. Among the 4,105 patients without syncope, 1,837 (44.8%) exhibited a positive response, including mixed type in 929 (22.6%) cases, cardioinhibitory type in 25 (0.6%) cases, vasodepressor type in 473 (11.5%) cases, POTS in 153 (3.7%) cases, and OH in 257 (6.3%) cases. Additionally, the positive rate of the HUTT in the syncope group was higher than that in non-syncope group (*P* < 0.001). Compared with the non-syncope group, a positive HUTT in the syncope group was significantly more frequent in the mixed type group (*P* < 0.01). There was no significant difference in the frequency of positive response to the HUTT between the syncope and non-syncope groups with the cardioinhibitory type (*P* = 0.085) and vasodepressor type (*P* = 0.058) (Table 1).

**Figure 1:**
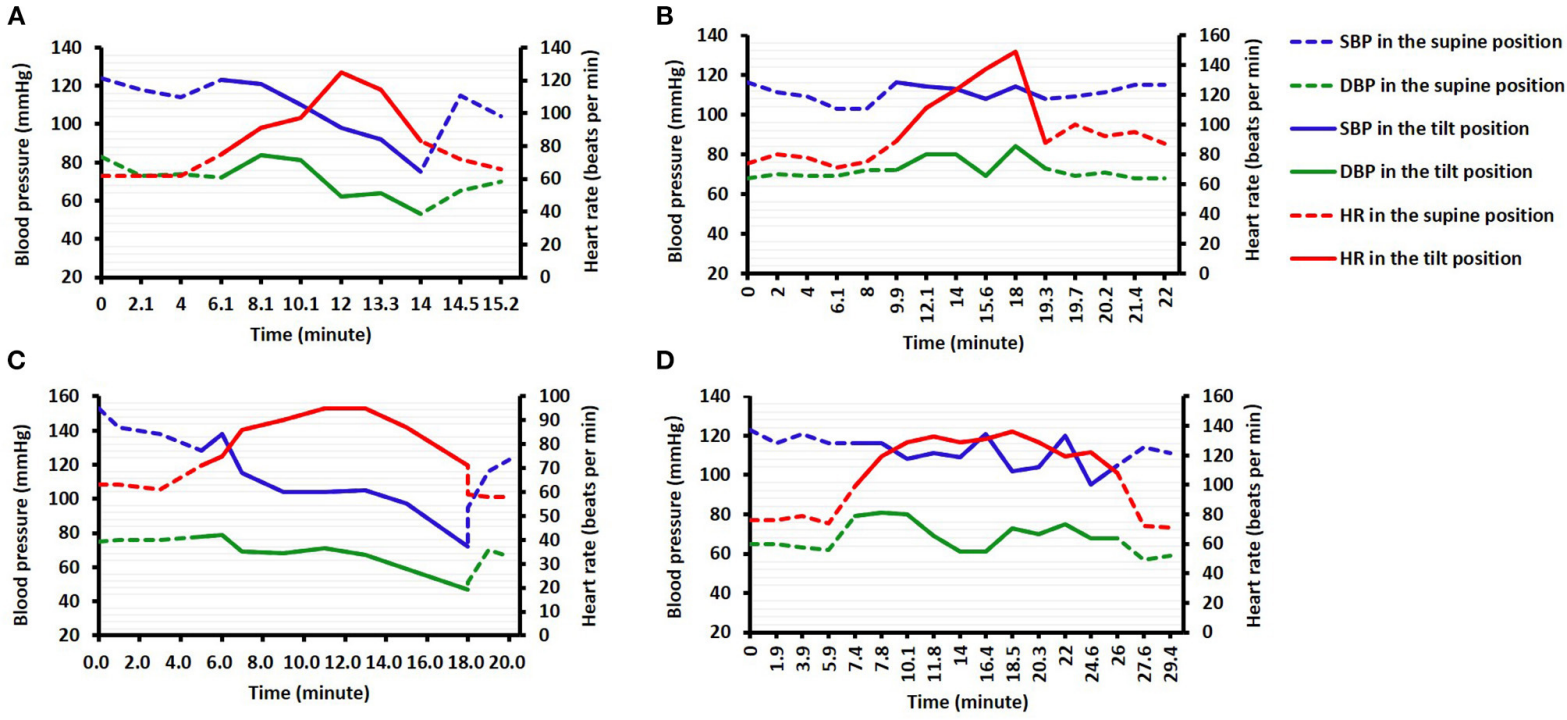
Changes in the maximum values of blood pressure (BP) and heart rate (HR) over time in patients with the mixed type **(A)**, cardioinhibitory type **(B)**, vasodepressor type **(C)**, and postural orthostatic tachycardia syndrome (POTS) **(D)**.

**Figure 2:**
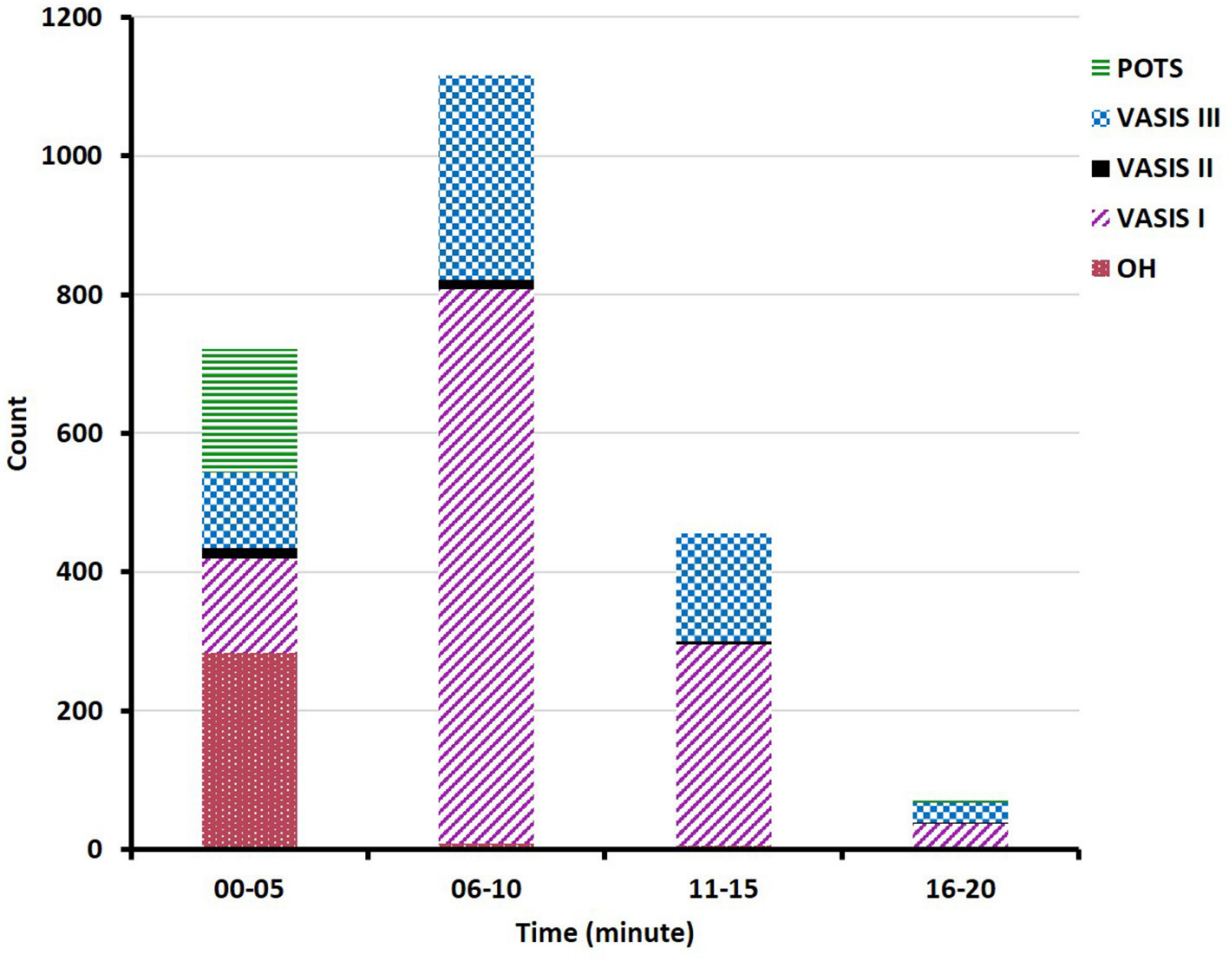
Bar graph showing the distribution of all of the positive types. POST, postural tachycardia syndrome; VASIS III, vasodepressor type; VASIS II, cardioinhibitory type; VASIS I, mixed type; OH, orthostatic hypotension.

Both the decreases in HR and SBP during the HUTT were similar between the syncope and non-syncope groups (Table 1). The study showed that there were changes in HR and BP prior to syncope or near-syncope symptoms, which presented as an increase in HR first, followed by successive decreases in BP and HR. The changes in HR and BP in the mixed-type and vasodepressor-type patients were as follows: the HR initially increased rapidly to the maximal HR when the patients had their head in the upward position, and the BP subsequently rapidly decreased to the lowest BP, followed by a decrease in HR. The changes in HR and BP in patients who had positive responses to the HUTT were described in Figure 1. A majority of positive responses (96.8%) were observed in the first 15 min of the HUTT, and the positive responses of POTS and OH mainly occurred in the first 5 min (Figure 2). These parameters usually returned to completely normal after 5 min of rest while the patient was in the supine position.

### Diagnostic Sensitivity of the HUTT

A total of 2,343 (48.1%) patients displayed a positive response. In other words, the overall sensitivity of the HUTT with pharmacological intervention was 48.1%. Among the patients in the syncope group, the sensitivity was 65.9% (52.6% in males and 77.9% in females), which was significantly higher than that in patients in the non-syncope group, with a sensitivity of 44.8% (36.5% in males and 50.6% in females) (*P* < 0.01). The sensitivity of the HUTT was higher in females than in males in both the syncope group (*P* < 0.01) and the non-syncope group (*P* < 0.01). Within the 4 age groups (<20, 21–40, 41–60, and >60 years old), the sensitivities were 74.7, 67.7, 45.6, and 31.2%, respectively. And all gender, age and symptom (whether suffer from a syncope or not) significantly affected the positive responses of HUTT (Table 2). Furthermore, in the non-syncope group, the sensitivities of the five subgroups, including the dizziness group, chest tightness/palpitation group, intermittent weakness group, sweating group, and intermittent unexplained arrhythmia group, were 38.6, 34.2, 44.6, 44.6, and 41.0%, respectively.

**Table 2:** Binary logistic regression analysis of related factors for positive HUTT results.

**Related factors**	**Wald value**	***P*-value**	**OR**	**95%CI**
Age	40.048	<0.001	0.989	0.986–0.992
**Gender**				
Male	336.483	<0.001	0.247	0.212–0.286
Female	–	–	–	–
**Symptom**				
Syncope	329.681	<0.001	6.223	5.116–7.595
Nonsyncope	–	–	–	–

*HUTT, head-up tilt test*.

### Safety and Adverse Events

The HRs that were detected during the HUTT ranged from 29 to 178 bpm, and patients whose HRs were <40 bpm accounted for 1.5% of the positive patients, 94.3% of whom had an isoprenaline-stimulated HUTT. The lowest SBP in this study was 54 mmHg, which was detected from the brachial artery. Patients who developed significant hypotension (could not be detected) accounted for 1.0% of the positive patients, and 95.7% of these patients had an isoprenaline-stimulated HUTT. There were two adverse events during the HUTT: one patient (60 years old, male) did not recover from the positive response after 2 min of rest in the supine position and had a junctional escape rhythm with significant hypotension (60–70/30–40 mmHg). Then, he recovered after 30 min following administration of 1 mg of intravenous atropine and an infusion of dopamine at a constant rate of 5–10 μg/kg/min. Another patient (65 years old, male) had asystole for 9 s, accompanied by loss of consciousness and hyperspasmia, but returned to the baseline value quickly after being placed in the supine position. However, none of these patients died during the HUTT process.

## Discussion

In this study, 4,873 patients with unexplained syncope or precursor syncope were enrolled. We found that the age of the syncope patients was younger than that of the non-syncope patients, and the proportion of males in the syncope group was higher. A positive HUTT was recorded in 48.1% of the study population, and the mixed type was the most frequently observed, followed by the vasodepressor type. Our study showed that the changes in HR and BP prior to syncope or near-syncope symptoms were proceeded by an increase in HR first, followed by a successive decrease in BP and HR. A majority of the positive responses were observed in the first 15 min of the HUTT. Our HUTT protocol had a higher sensitivity in young females with syncope. Among the non-syncope population, patients who presented with weakness and sweating more frequently displayed a positive response (higher sensitivity). There were no serious adverse events or deaths during the HUTT process.

Compared with precursor syncope patients, the patients with syncope were younger, which showed that there was more exaggerated vagal activity in the younger patients. This result was supported by a report from Noormand et al. that showed that the overexcitation of vagal activity became less frequent with age (13). We also found that the proportion of females included in the non-syncope group was higher, the reasons for which were as follows. Precursor syncope symptoms, such as dizziness, chest tightness, palpitation, and weakness, were subjective and non-specific and may be associated with anxiety and depression. Previous studies have shown that females were more likely than males to suffer from anxiety and depression (14, 15), which probably led to inappropriate HUTT performance, which decreased the sensitivity of the HUTT in our study. Moreover, the proportion of patients with syncope who visited other departments initially was higher than that of the non-syncope patients, which may be attributed to the presence of wounds that occurred secondary to syncope, and this helps to remind surgeons that they should be cautious of VVS when they see patients with wounds secondary to syncope.

Of the positive patients, the mixed type was the most frequently observed in our study, which was similar to the result of a previous study (16). And the previous study also suggested that the cardioinhibitory type was the second most frequently observed, which was different from our result (vasodepressor type). A possible reason was that the patients in our study were older (48.3 ± 17.6 vs. 34.0 ± 11.2 years), as the cardioinhibitory response decreases with age (13, 16). Our study revealed that the HR initially increased in patients identified as having VVS during the HUTT, followed by successive decreases in BP and HR. This regular pattern seemed to be due to a series of autonomic changes as reported in a previous paper (17), namely, increased excitability of the sympathetic nerves, decreased excitability of the sympathetic nerves, and increased excitability of the parasympathetic nerves. Based on the pathophysiological mechanism, many drugs, such as β-blockers, scopolamine, and etilefrine, have been tested for the treatment of VVS (18). In addition, we also found that a majority of positive responses were observed in the first 15 min of the HUTT, demonstrating that the time of observation during the HUTT was reasonable. This result serves as a reminder that more attention should be paid within the first 15 min during the HUTT.

The sensitivity of the HUTT has been reported to range from 46 to 65% for glyceryl trinitrate and 21 to 65% for isoprenaline (16, 19–23). Among the patients in the syncope group, the sensitivity was 65.9% in our study, which is higher than that reported previously. This result could be explained by the followings. First, any nitroglycerin-potentiated HUTT or isoprenaline-potentiated HUTT with a positive response was identified as a positive response in our study, which could lead to an increase in the sensitivity. Second, the patients were evaluated by a thorough evaluation, including an electrocardiogram, ECG, echocardiography, electroencephalogram, and cranial CT/MRI, which helped exclude the presence of cardiogenic syncope and neurological disease. In other words, the patients we included were suspected to have significant VVS. In addition, there was a higher sensitivity in young females, which was similar to the results reported by previous studies (13, 24). In this study, we also included precursor syncope patients, approximately half of whom displayed a positive response, suggesting that it is necessary to perform a HUTT for patients with precursor syncope who do not have evidence of cardiogenic disease or neurological disease, especially young females that have weakness and sweating. This probably resulted in a decrease in the numbers of misdiagnoses and missed diagnoses.

The HUTT protocol that we adopted was safe, as few serious adverse events were encountered with either protocol, which was similar to the results from previous studies revealing that many patients tolerated glyceryl trinitrate well. However, some patients tolerated isoprenaline poorly (16, 21, 22). The underlying mechanism that makes patients tolerate isoprenaline poorly is as follows. Isoprenaline exaggerates sympathetic nerve activity, which causes a significant decrease in the left ventricular volume, inducing myocardial ischemia and hypotension (22, 25). Graham et al. showed that there was an association between older age and a high adverse event rate. Therefore, physicians must be cautious of older patients with cardiovascular comorbidities when an isoprenaline-potentiated HUTT is performed. In this study, two patients over 60 years of age suffered from an adverse event, which may be attributed to cardiovascular comorbidities.

## Limitations

There were several limitations in our study. First, any nitroglycerin-potentiated HUTT or isoprenaline-potentiated HUTT presenting as a positive response was identified as a positive response in our study, which may have caused an increase in the sensitivity and a decrease in the specificity. A previous paper revealed that the specificities varied between 71 and 94.7% for glyceryl trinitrate and between 64 and 89.4% for isoprenaline (16, 20, 22). However, we could not determine the specificity of the HUTT protocol, as no healthy subjects were included in our study. Second, there is no gold standard test for the diagnosis of VVS, and the positive response rate of the HUTT, which was used to calculate the sensitivity, cannot reflect the real sensitivity of the HUTT. Thus, the false-positive and false-negative rates of the HUTT could not be acquired. Third, many patients with precursor syncope were included in this study and underwent a HUTT, which may lead to an excessive use of the HUTT. More studies are needed to identify the clinical characteristics of precursor syncope patients who need to undergo a HUTT.

## Conclusion

The direct drug-potentiated HUTT is a feasible substitution for the conventional HUTT and has a high sensitivity and low risk. Young females presenting with near-syncope symptoms, especially weakness and sweating, who are highly suspicious of having VVS (although not suffering from syncope), should undergo a HUTT to decrease the chance for a misdiagnosis or a missed diagnosis.

## Data Availability Statement

The raw data supporting the conclusions of this article will be made available by the authors, without undue reservation.

## Ethics Statement

The studies involving human participants were reviewed and approved by Institutional Ethics Committee of The Xianyang Central Hospital. Written informed consent to participate in this study was provided by the participants' legal guardian/next of kin. Written informed consent was obtained from the minor(s)' legal guardian/next of kin for the publication of any potentially identifiable images or data included in this article.

## Author Contributions

LX, FG, and CS: proposal. LX and XC: drafting. RW, YD, YY, JH, JW, BC, XX, and BZ: data gathering. HM, LX, FG, and CS: supervision and revision. LX: statistical analysis. All authors approved the final draft.

## Conflict of Interest

The authors declare that the research was conducted in the absence of any commercial or financial relationships that could be construed as a potential conflict of interest.

## Publisher's Note

All claims expressed in this article are solely those of the authors and do not necessarily represent those of their affiliated organizations, or those of the publisher, the editors and the reviewers. Any product that may be evaluated in this article, or claim that may be made by its manufacturer, is not guaranteed or endorsed by the publisher.
